# Combination therapy of capecitabine, irinotecan, oxaliplatin, and bevacizumab as a first‐line treatment for metastatic colorectal cancer: Safety lead‐in results from the QUATTRO-II study

**DOI:** 10.1007/s10637-021-01125-2

**Published:** 2021-05-21

**Authors:** Daisuke Kotani, Takayuki Yoshino, Masahito Kotaka, Akihito Kawazoe, Toshiki Masuishi, Hiroya Taniguchi, Kentaro Yamazaki, Takeharu Yamanaka, Eiji Oki, Kei Muro, Yoshito Komatsu, Hideaki Bando, Hironaga Satake, Takeshi Kato, Akihito Tsuji

**Affiliations:** 1 Department of Gastroenterology and Gastrointestinal Oncology, National Cancer Center Hospital East, 6-5-1 Kashiwanoha, Kashiwa City, Chiba 277-8577 Japan; 2 Gastrointestinal Cancer Center, Sano Hospital, Kobe, Japan; 3 Department of Clinical Oncology, Aichi Cancer Center Hospital, Nagoya, Japan; 4 Division of Gastrointestinal Oncology, Shizuoka Cancer Center, Shizuoka, Japan; 5 Department of Biostatistics, Yokohama City University School of Medicine, Yokohama, Japan; 6 Department of Surgery and Science, Graduate School of Medical Sciences, Kyushu University, Fukuoka, Japan; 7 Department of Cancer Chemotherapy, Hokkaido University Hospital Cancer Center, Sapporo, Japan; 8 Cancer Treatment Center, Kansai Medical University Hospital, Hirakata City, Japan; 9 Department of Surgery, National Hospital Organization Osaka National Hospital, Osaka, Japan; 10 Department of Medical Oncology, Kagawa University Hospital, 1750-1 Ikenobe, Kita District, Miki, Kagawa 761-0793 Japan

**Keywords:** Metastatic colorectal cancer, CAPOXIRI, Triplet, Bevacizumab, FOLFOXIRI

## Abstract

*Background *FOLFOXIRI plus bevacizumab is the first-line treatment for metastatic colorectal cancer (mCRC) but demonstrates high neutropenia incidence among Asian patients. Hence, we conducted the randomized phase II QUATTRO-II study (ClinicalTrials.gov identifier: NCT04097444; Japan Registry of Clinical Trials identifier: jRTCs041190072) to evaluate the safety and efficacy of capecitabine, oxaliplatin, and irinotecan (CAPOXIRI) combination plus bevacizumab versus FOLFOXIRI plus bevacizumab, expecting a lower incidence of neutropenia without compromising the efficacy. *Methods *We investigated the recommended doses (RD) of oxaliplatin and irinotecan as a safety lead-in portion of Step 1 before initiating the randomized portion as Step 2. Four dose levels of CAPOXIRI (fixed dose of capecitabine, 1600 mg/m^2^; escalated/de-escalated doses of oxaliplatin and irinotecan) plus bevacizumab (7.5 mg/kg) were investigated in a 3 + 3 manner. A dose level of ≤ 2/6 of dose-limiting toxicity (DLT) cases was expected as the RD. *Results *In Step 1, we included nine patients (three and six in levels 0 and + 1, respectively). Level 0 (irinotecan, 200 mg/m^2^; oxaliplatin, 100 mg/m^2^) did not demonstrate DLTs. In level + 1 (irinotecan, 200 mg/m^2^; oxaliplatin, 130 mg/m^2^), although one patient experienced grade 4 febrile neutropenia, no further safety concerns were observed. As a preliminary efficacy result, the objective response rate in all nine patients was 89 % (100 and 83 % in levels 0 and + 1, respectively). *Conclusions *The RD of CAPOXIRI plus bevacizumab was 200, 130, and 1600 mg/m^2^ for irinotecan, oxaliplatin, and capecitabine, respectively, and 7.5 mg/kg for bevacizumab. The randomized portion is still ongoing.

**Supplementary Information:**

The online version contains supplementary material available at 10.1007/s10637-021-01125-2.

## Introduction

Metastatic colorectal cancer (mCRC) has several treatment options for its first-line treatment [1–3]. The phase III TRIBE study demonstrated that fluorouracil, leucovorin, oxaliplatin, and irinotecan (FOLFOXIRI) combination plus bevacizumab has better progression-free survival (PFS), response rate (RR), and overall survival (OS) than fluorouracil, leucovorin, and irinotecan (FOLFIRI) combination plus bevacizumab, as a first-line treatment of mCRC [4]. More recently, the phase III TRIBE2 study revealed that the primary endpoint of PFS2, which is the time from randomization to disease progression on any treatment given after first disease progression, or death, is significantly longer in FOLFOXIRI plus bevacizumab than in the first-line FOLFOX (fluorouracil, leucovorin, and oxaliplatin) plus bevacizumab followed by FOLFIRI plus bevacizumab after disease progression [5]. Therefore, FOLFOXIRI plus bevacizumab is a valuable first-line treatment option. However, despite the significant survival benefit of FOLFOXIRI plus bevacizumab, the incidence of grade 3 or 4 adverse events, including neutropenia (50 %), diarrhea (18.8 %), and stomatitis (8.8 %), is increased, raising a concern if applied in clinical practice [4]. Furthermore, irinotecan-based regimen tends to cause a higher incidence of grade 3 or 4 neutropenia in Asian patients than in Caucasian patients [6, 7]. Indeed, in the single-arm phase II QUATTRO study, which assessed the safety and efficacy of FOLFOXIRI plus bevacizumab in Japanese population, the incidence of grade 3 or 4 neutropenia (72.5 %) and febrile neutropenia (21.7 %) were relatively high [8].

In the Asian phase III AXEPT study, the combination of modified capecitabine (1600 mg/m^2^) and irinotecan (200 mg/m^2^) (CAPIRI) plus bevacizumab (7.5 mg/kg) has a longer primary endpoint of OS as a second-line treatment and a lower incidence of hematologic toxicity than FOLFIRI plus bevacizumab [6]. Moreover, the phase II AIO0604 study demonstrated that the PFS and OS of modified CAPIRI plus bevacizumab are similar to those of CAPOX plus bevacizumab as a first-line treatment [9]. Thus, the reduced dose of capecitabine in combination with irinotecan and oxaliplatin (CAPOXIRI) plus bevacizumab might be more feasible than FOLFOXIRI plus bevacizumab as a first-line treatment, without compromising the efficacy.

The QUATTRO-II study is an open-label, randomized, phase II study that evaluates the efficacy and safety of CAPOXIRI plus bevacizumab versus FOLFOXIRI plus bevacizumab as a first-line treatment of mCRC [10]. Before the randomized portion (Step 2), the recommended doses (RD) of CAPOXIRI plus bevacizumab were investigated as a safety lead-in in Step 1. Here, we describe the results of Step 1 in the QUATTRO-II study.

## Materials and methods

The main inclusion criteria were the following: ≥20 years of age; unresectable colorectal adenocarcinoma with measurable lesions according to Response Evaluation Criteria in Solid Tumors (RECIST) version 1.1 [11]; Eastern Cooperative Oncology Group performance status (ECOG PS) of 0 or 1 (only PS 0 was included in patients aged ≥ 71 years); *RAS*/*BRAF* status diagnosed as either wild type or mutant; wild type (*UGT1A1* *1/*1) or single heterozygous type (*1/*6 or *1/*28) of *UGT1A1* polymorphism; adequate organ function; and no chemotherapy history. Online Resource 1: Table S1 lists additional inclusion and exclusion criteria for this study.

Eight institutions in Japan participated in Step 1. The study was conducted in accordance with Clinical Trials Act (Act No. 16 of April 14, 2017) in Japan, as well as with the ethical guidelines for medical and health research involving human subjects. Written informed consent was obtained in all patients (ClinicalTrials.gov identifier: NCT04097444; Japan Registry of Clinical Trials identifier: jRTCs041190072).

### Study procedures

The dose schedule of CAPOXIRI plus bevacizumab was as follows: bevacizumab (7.5 mg/kg) infusion for 30–90 min, irinotecan infusion for 1 h, oxaliplatin infusion for 2 h, and capecitabine (1600 mg/m^2^/day) for 1–14 days every 3 weeks. In dose escalation or de-escalation analysis, the following four levels of CAPOXIRI doses were investigated by including every three patients: irinotecan (200 mg/m^2^) and oxaliplatin (130 mg/m^2^) for level + 1; irinotecan (200 mg/m^2^) and oxaliplatin (100 mg/m^2^) for level 0; irinotecan (180 mg/m^2^) and oxaliplatin (100 mg/m^2^) for level − 0.5; and irinotecan (150 mg/m^2^) and oxaliplatin (100 mg/m^2^) for level − 1. The starting dose was level 0. CAPOXIRI plus bevacizumab was administered for up to six cycles (maximum eight cycles), followed by the maintenance of capecitabine plus bevacizumab or 5-FU/l-LV plus bevacizumab by investigator’s discretion until disease progression or unacceptable toxicities occurred.

### Statistical methods

For the safety and efficacy analyses, we included all patients who received at least one dose of the study treatment. Adverse events were graded according to the National Cancer Institute Common Terminology Criteria for Adverse Events (CTCAE) version 5.0 [12]. The endpoint of Step 1 was to assess the safety and decide the RD of the study treatment. We used a 3 + 3 dose-escalation or de-escalation design; if no dose-limiting toxicities (DLTs) were recorded in the first treatment cycle, the doses were escalated to the next level in the additional three patients. DLTs in the first cycle were defined as follows: grade 4 neutropenia over 8 days; febrile neutropenia; grade 4 thrombocytopenia or grade 3 thrombocytopenia requiring platelet transfusion; and grade 3 digestive symptoms that did not improve after ≥ 5 days despite optimal treatment. If DLTs occurred in one patient during the first cycle, three additional patients would be treated at that dose level. Prophylactic granulocyte-colony stimulating factor (G-CSF) was prohibited. For Step 2 as the randomized phase II part, the steering committee would determine the RD.

Treatment response and disease progression were radiologically assessed by computed tomography (CT) scanning based on RECIST version 1.1. CT was then evaluated once every 8 weeks for the first 72 weeks and then every 12 weeks.

## Results

Between November 2019 and March 2020, nine patients with mCRC were enrolled in Step 1 of the QUATTRO-II study. As of September 18, 2020, study treatment was ongoing in four patients. Meanwhile, five patients discontinued the study treatment because of conversion surgery for metastases and/or primary tumor (n = 3), disease progression (n = 1), and toxicity (n = 1).

### Patient characteristics

Table 1 summarizes the baseline characteristics of the eligible patients. The median age was 62 (45–78) years, and the ECOG PS was 0 in eight patients (89 %). Eight patients (89 %) had two or more metastatic sites, and six patients (67 %) had synchronous metastatic disease. In addition, five patients (56 %) underwent primary tumor resection. Only one patient had received previous adjuvant chemotherapy of capecitabine monotherapy. Seven patients and two patients were *RAS* mutant and *RAS* wild-type, respectively. No *BRAF* V600E mutation was detected. For the *UGT1A1* genotype, seven patients had *1/*1, while two patients had *1/*28. Baseline carcinoembryonic antigen (CEA) was higher than upper limit of normal in seven patients.

**Table 1:** Baseline patient characteristics

Characteristics		N = 9 (%)
Median, age (range)		62 (45–78)
Gender	Male	6 (67)
	Female	3 (33)
ECOG performance status	0	8 (89)
	1	1 (11)
Primary tumor location	Right colon	3 (33)
	Left colon or rectum	6 (67)
Surgery for primary tumor	Yes	5 (56)
Previous adjuvant chemotherapy	Yes	1 (11)
Time to metastases	Synchronous	6 (67)
	Metachronous	3 (33)
Disease site (overlapped)	Liver	6 (67)
	Lymph nodes	6 (67)
	Lung	5 (56)
	Peritoneum	3 (33)
	Bone	1 (11)
Number of metastatic sites	< 2	1 (11)
	≥ 2	8 (89)
*UGT1A1* genotype	*1/*1	7 (78)
	*1/*28	2 (22)
*RAS*/*BRAF* status	*RAS* mutant	7 (78)
	*RAS*/*BRAF* wild type	2 (22)
	*BRAF* V600E mutant	0 (0)
CEA	> ULN*	7 (78)
	≤ ULN	2 (22)

* ULN; Upper limit of normal

### Safety

#### DLTs

One of the three patients in level 0 (irinotecan, 200 mg/m^2^; oxaliplatin, 100 mg/m^2^) manifested grade 4 neutropenia and grade 3 anorexia, but both adverse events recovered immediately, thereby not conflicting with the DLT criteria in level 0. In level + 1 (irinotecan, 200 mg/m^2^; oxaliplatin, 130 mg/m^2^), only one of the six patients exhibited grade 4 febrile neutropenia. In addition, grade 3 colitis and grade 4 neutropenia occurred in one and two patients, respectively, within the first cycle. With appropriate supportive care, all treatment-related toxicities were resolved, with no treatment-related death. No further safety concerns occurred in the subsequent cycles. Therefore, the steering committee inferred that the doses in level + 1 were the RDs for Step 2.

#### Adverse events

Table 2 lists the treatment-related adverse events in all nine patients during study treatment. Two patients with grade 4 neutropenia received G-CSF. In two patients with *UGT1A1* *1/*28, one experienced grade 4 neutropenia, while the other had no grade 3 or higher hematological toxicities. The most frequent grade 3 or 4 treatment-related adverse events among the six patients in level + 1 of the RD were neutropenia (n = 3, 50 %), leukopenia (n = 2, 33 %), fatigue (n = 1, 17 %), hypertension (n = 1, 17 %), colitis (n = 1, 17 %), and febrile neutropenia (n = 1, 17 %). Meanwhile, four of six patients experienced treatment delay ≥ 4 days because of investigator’s judgment (n = 2), febrile neutropenia (n = 1), and patient convenience (n = 1). In the second or subsequent cycles, five of six patients required dose reduction in at least one study drug because of neutropenia (n = 2), febrile neutropenia (n = 1), fatigue (n = 1), and investigator’s judgment (n = 1).

**Table 2:** Treatment-related adverse events

	All (n = 9)				Level 0 (n = 3)				Level + 1 (n = 6)			
Adverse events, N (%)	All grades		≥Grade 3		All grades		≥Grade 3		All grades		≥Grade 3	
All events	9	(100)	7	(78)	3	(100)	2	(67)	6	(100)	5	(83)
Hematology												
Neutropenia	4	(44)	4	(44)	1	(33)	1	(33)	3	(50)	3	(50)
Leukopenia	3	(33)	2	(22)	0	(0)	0	(0)	3	(50)	2	(33)
Anemia	1	(11)	0	(0)	0	(0)	0	(0)	1	(17)	0	(0)
Thrombocytopenia	1	(11)	0	(0)	0	(0)	0	(0)	1	(17)	0	(0)
Nonhematology												
Anorexia	9	(100)	1	(11)	3	(100)	1	(33)	6	(100)	0	(0)
Diarrhea	7	(78)	0	(0)	2	(67)	0	(0)	5	(83)	0	(0)
Nausea	6	(67)	0	(0)	3	(100)	0	(0)	3	(50)	0	(0)
Peripheral sensory neuropathy	6	(67)	0	(0)	2	(67)	0	(0)	4	(67)	0	(0)
Fatigue	3	(33)	1	(11)	1	(33)	0	(0)	2	(33)	1	(17)
Hypertension	3	(33)	1	(11)	1	(33)	0	(0)	2	(33)	1	(17)
Alopecia	3	(33)	0	(0)	2	(67)	0	(0)	1	(17)	0	(0)
Colitis	2	(22)	1	(11)	0	(0)	0	(0)	2	(33)	1	(17)
Dehydration	2	(22)	1	(11)	1	(33)	1	(33)	1	(17)	0	(0)
Abdominal pain	2	(22)	0	(0)	0	(0)	0	(0)	2	(33)	0	(0)
Mucositis oral	2	(22)	0	(0)	0	(0)	0	(0)	2	(33)	0	(0)
Malaise	2	(22)	0	(0)	0	(0)	0	(0)	2	(33)	0	(0)
Proteinuria	2	(22)	0	(0)	0	(0)	0	(0)	2	(33)	0	(0)
Bleeding	2	(22)	0	(0)	1	(33)	0	(0)	1	(17)	0	(0)
Febrile neutropenia	1	(11)	1	(11)	0	(0)	0	(0)	1	(17)	1	(17)

#### Drug discontinuations

At least one study drug was discontinued in three patients because of adverse events. All study drugs were discontinued in one patient with grade 4 febrile neutropenia, oxaliplatin was discontinued in one patient with grade 2 peripheral sensory neuropathy, and bevacizumab was discontinued in one patient with vein thrombosis.

### Efficacy

**Fig. 1:**
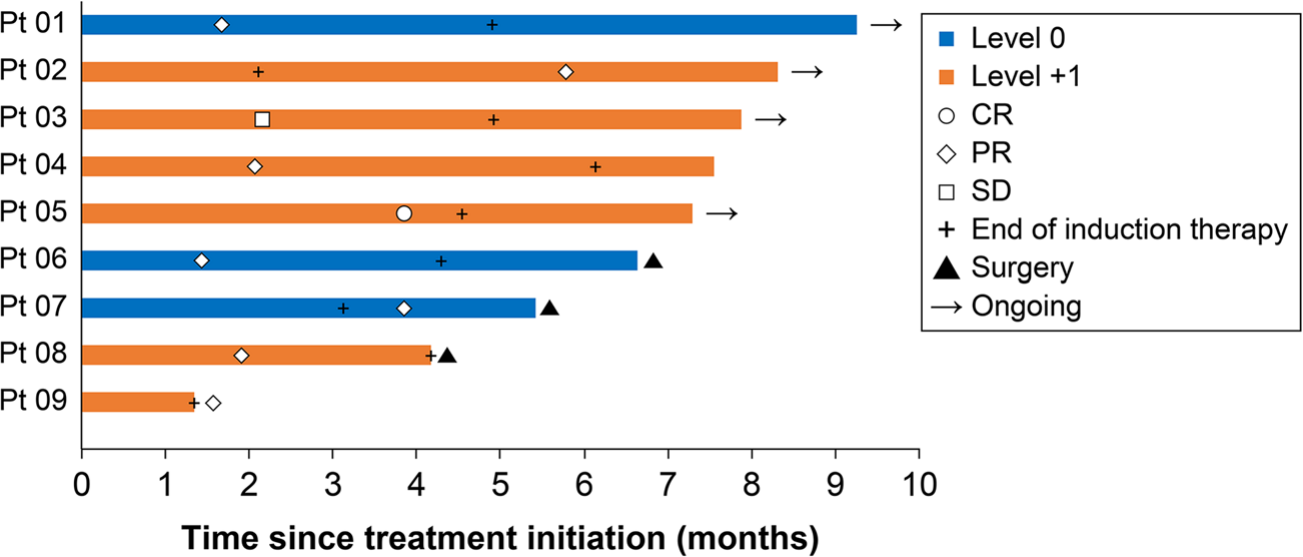
Swimmer plots according to dose level. Study treatment was ongoing in four patients (Pt 01, Pt 02, Pt 03, Pt 05). Study treatment was discontinued due to conversion surgery (Pt 06, Pt 07, Pt 08), disease progression (Pt 04), and toxicity (Pt 09)

**Fig. 2:**
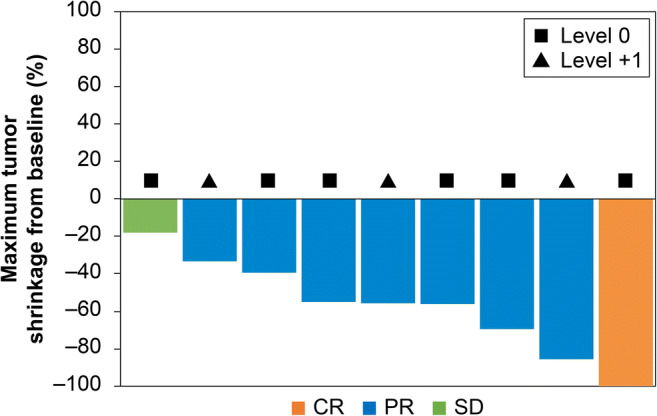
Waterfall plot of the maximum percent change in tumor size from baseline, as measured according to Response Evaluation Criteria in Solid Tumors

Objective response was observed in eight patients in which one and seven patients exhibited complete and partial responses, respectively (Table 3). The objective response rate (ORR) was 89 % in all nine patients (level 0, 100 %; level + 1, 83 %). Meanwhile, the one remaining patient had a stable disease with 18 % tumor shrinkage, resulting in a disease control rate of 100 %. Five (63 %) of the responding patients achieved a response within 2 months, seven (88 %) within 4 months, and all within 6 months from study enrollment. All three patients who underwent conversion surgery for metastases and/or primary tumor achieved a partial response within 4 months (Fig. 1). Figure 2 presents the tumor measurement changes from baseline.

**Table 3:** Best overall response

Dose level	All	Level 0	Level + 1
	n = 9 (%)	n = 3 (%)	n = 6 (%)
Complete response (CR)	1 (11)	0 (0)	1 (17)
Partial response (PR)	7 (78)	3 (100)	4 (67)
Stable disease (SD)	1 (11)	0 (0)	1 (17)
Progressive disease (PD)	0 (0)	0 (0)	0 (0)
Objective response rate (CR + PR)	8 (89)	3 (100)	5 (83)
Disease control rate (CR + PR + SD)	9 (100)	3 (100)	6 (100)

## Discussion

In this study, we evaluated the RD, safety, and efficacy of CAPOXIRI plus bevacizumab as a first-line treatment for mCRC. The RD was determined to be 200 mg/m^2^ for irinotecan, 130 mg/m^2^ for oxaliplatin, 1600 mg/m^2^/day for capecitabine, and 7.5 mg/kg for bevacizumab every 3 weeks. In the previous dose-escalation study, the RD was 150 mg/m^2^ for irinotecan, 100 mg/m^2^ for oxaliplatin, 1700 mg/m^2^/day for capecitabine, and 7.5 mg/kg for bevacizumab every 3 weeks; however, no DLT was observed in the maximum dose of irinotecan (150 mg/m^2^); thus, the true maximum tolerated dose was not reached [13]. In our study, we could escalate the dose of irinotecan (200 mg/m^2^) as well as oxaliplatin (130 mg/m^2^) in combination with capecitabine (1600 mg/m^2^/day) and bevacizumab (7.5 mg/kg).

The toxicities in CAPOXIRI plus bevacizumab were generally well tolerated. Notably, considering that irinotecan-based regimens including FOLFIRI plus bevacizumab or FOLFOXIRI plus bevacizumab have a high incidence of grade 3 or higher neutropenia among Asian patients [6–8], the frequency of grade 3 or higher neutropenia (50 %) in the RD tended to be lower and feasible in the current study. According to *UGT1A1* genotype, grade 4 neutropenia and febrile neutropenia occurred in 46.2 and 25.6 %, respectively, of patients with *UGT1A1* *1/*6 or *1/*28 single heterozygous type in the previous QUATTRO study [8]. Although only two patients with *UGT1A1* single heterozygous type were enrolled in this study, one of them experienced grade 4 neutropenia. The results from the previous QUATTRO study and this study suggest that patients receiving CAPOXIRI plus bevacizumab as well as FOLFOXIRI plus bevacizumab must be carefully observed, especially during the first cycle.

The ORR of CAPOXIRI was 89 %, indicating that its efficacy is promising as a first-line treatment for patients with mCRC, albeit the preliminary and small sample size study. In addition, five of the responding patients achieved a response within 2 months and six patients achieved ≥ 50 % maximum tumor shrinkage from baseline, resulting in conversion surgery in three patients. Although our data were immature with a shorter follow-up period, the dose-escalation strategy of irinotecan and oxaliplatin with a modified dose of capecitabine might have a higher antitumor activity and a deeper response.

The randomized portion (Step 2) of the QUATTRO-II study is based on the results in Step 1, and it is still ongoing [10]. Step 2 aims to evaluate the similarity between CAPOXIRI plus bevacizumab and FOLFOXIRI plus bevacizumab as a first-line treatment in patients with mCRC. If CAPOXIRI plus bevacizumab is confirmed to have manageable toxicities, including neutropenia, with similar efficacy to FOLFOXIRI plus bevacizumab, CAPOXIRI plus bevacizumab might potentially become a new treatment option as a first-line treatment for mCRC.

## Supplementary Information

ESM 1 (PDF 441 KB)

ESM 2 (DOC 219 KB)

## Data Availability

The data that support the findings of this study are available from the corresponding author, upon reasonable request.
