# Supplementary material for: Combination therapy of capecitabine, irinotecan, oxaliplatin, and bevacizumab as a first‐line treatment for metastatic colorectal cancer: Safety lead‐in results from the QUATTRO-II study
Source: Invest New Drugs. 2021 May 21;39(6):1649–55. doi: 10.1007/s10637-021-01125-2 (PMC8541955; doi:10.1007/s10637-021-01125-2)
Supplement: Supplementary file 1 — (PDF 441 KB) [file 10637_2021_1125_MOESM1_ESM.pdf]

Online Resource 1: Table S1: Patient inclusion and exclusion criteria

Inclusion criteria
1. Provision of personally written informed consent after receiving full explanation of the study
2. Histologically confirmed colon or rectal adenocarcinoma (excluding appendix cancer and anal canal cancer)
3. Clinically unresectable tumor
4. ≥20 years of age at enrollment
5. ECOG performance status (PS) score of 0 or 1 (≥71 years of age: PS score of 0)
6. Measurable lesion in accordance with RECIST version 1.1 criteria on contrast-enhanced computed tomography of the chest, abdominal, or pelvic (trunk) (required within 28 days of enrollment)
7. No previous chemotherapy for colon or rectal cancer (only patients with confirmed relapse ≥ 24 weeks after completion of postoperative adjuvant chemotherapy can be enrolled)
8. *RAS*/*BRAF* mutation analysis at enrollment identifies *RAS*/*BRAF* status as either the wild type or mutant type
9. Vital organ functions meet the following criteria within 14 days before enrollment.
If multiple test results are available in that period, the results closest to the enrollment date will be used. No blood transfusions or hematopoietic factor administration will be permitted within 2 weeks before the measurement date.
a. Neutrophil count: ≥1500/mm^3
b. Platelet count: ≥10.0 × 10^4/mm^3
c. Hemoglobin concentration: ≥9.0 g/dL
d. Total bilirubin: ≤1.5-fold of the upper limit of normal (ULN)
e. AST, ALT, ALP: ≤2.5-fold of the ULN (≤5-fold of the ULN for liver metastases)
f. Serum creatinine: ≤1.5-fold of the ULN, or creatinine clearance: ≥30 mL/min
g. Urine protein: ≤2+ (if ≥3+, urine protein/creatinine ratio: <2.0)
10. *UGT1A1* polymorphism is a wild type or a single heterozygous type

Exclusion criteria
1. Previous radiation therapy in which ≥20% of bone marrow was exposed to the radiation field
2. Untreated brain metastases, spinal cord compression, or primary brain tumor
3. History of central nervous system disease (excluding asymptomatic lacunar infarction)

4. Requirement of continuous systemic corticosteroid treatment

5. Inconsistently controlled oral or parenteral anticoagulant (e.g., low-molecular-weight heparin) dose (≥14 days; oral anticoagulants: conditions with high bleeding risk, such as PT-INR ≥ 3 and clinically significant active bleeding [within 14 days of enrollment])

6. Arterial thrombosis or arterial thromboembolism such as myocardial infarction, transient ischemic attack, or cerebrovascular attack in the last year before enrollment

7. Previous treatment using an investigational drug within 28 days before enrollment, or participation in a study of an unapproved drug

8. Any of the following comorbidities:

   a. Uncontrolled hypertension

   b. Uncontrolled diabetes mellitus

   c. Uncontrolled diarrhea

   d. Peripheral sensory neuropathy (≥Grade 1)

   e. Active peptic ulcer

   f. Unhealed wound (except for suturing associated with implanted port placement)

   g. Evidence of cardiovascular disease, cerebrovascular disorder (within 24 weeks), myocardial infarction (within 24 weeks), unstable angina pectoris, New York Hearth Association classification ≥Grade 2 congestive heart failure, serious arrhythmias requiring drug therapy

   h. Uncontrolled venous thromboembolism (unless clinically stable, asymptomatic, or appropriately treated with an anticoagulant)

   i. Systemic treatment required for, or evidence of, infections

   j. Other clinically significant diseases (e.g., interstitial pneumonia or renal impairment)

9. Major surgical procedure within 28 days before study treatment initiation

10. Physical defects of the upper gastrointestinal tract; malabsorption syndrome or difficulty taking oral medication

11. Pregnant, breastfeeding, positive pregnancy test (women who have menstruated in the last year will be tested), or patients who are unwilling to use contraception during the study

12. Active hepatitis B or C, or evidence of HIV infection

13. Previous chemotherapy for other malignancies (excluding hormone therapy for breast cancer).

14. Other active malignancies (excluding malignancies that are expected to be completely cured, such as intramucosal carcinoma and carcinoma *in situ*)

15. Diseases such as intestinal paralysis, intestinal obstruction, or gastrointestinal perforation within 1 year before enrollment

16. Pleural effusion, ascites, or pericardial effusion requiring drainage

17. History of hypersensitivity to fluorouracil, levofolinate, oxaliplatin, irinotecan, bevacizumab, and their excipients or Chinese hamster ovary cell proteins

18. History of adverse reactions to fluoropyrimidine drugs indicative of dihydropyrimidine dehydrogenase (DPD) deficiency

19. Endoluminal stenting

20. Unsuitable for the study according to the opinion of the investigators
